# Factors Associated With Adoption of the Electronic Health Record System Among Primary Care Physicians

**DOI:** 10.2196/medinform.2766

**Published:** 2013-08-26

**Authors:** Clement SK Cheung, Ellen LH Tong, Ngai Tseung Cheung, Wai Man Chan, Harry HX Wang, Mandy WM Kwan, Carmen KM Fan, Kirin QL Liu, Martin CS Wong

**Affiliations:** ^1^Hospital Authority Information Technology ServicesHealth Informatics SectionHong KongChina (Hong Kong); ^2^School of Public Health and Primary CareThe Chinese University of Hong KongShatin, NT, Hong KongChina (Hong Kong); ^3^General Practice and Primary CareInstitute of Health and WellbeingUniversity of GlasgowGlasgowUnited Kingdom

**Keywords:** electronic medical record, physicians, adoption, associated factors, medical informatics

## Abstract

**Background:**

A territory-wide Internet-based electronic patient record allows better patient care in different sectors. The engagement of private physicians is one of the major facilitators for implementation, but there is limited information about the current adoption level of electronic medical record (eMR) among private primary care physicians.

**Objective:**

This survey measured the adoption level, enabling factors, and hindering factors of eMR, among private physicians in Hong Kong. It also evaluated the key functions and the popularity of electronic systems and vendors used by these private practitioners.

**Methods:**

A central registry consisting of 4324 private practitioners was set up. Invitations for self-administered surveys and the completed questionnaires were sent and returned via fax, email, postal mail, and on-site clinic visits. Current users and non-users of eMR system were compared according to their demographic and practice characteristics. Student’s *t* tests and chi-square tests were used for continuous and categorical variables, respectively.

**Results:**

A total of 524 completed surveys (response rate 524/4405 11.90%) were collected. The proportion of using eMR in private clinics was 79.6% (417/524). When compared with non-users, the eMR users were younger (users: 48.4 years SD 10.6 years vs non-users: 61.7 years SD 10.2 years, *P*<.001); more were female physicians (users: 80/417, 19.2% vs non-users: 14/107, 13.1%, *P*=.013); possessed less clinical experience (with more than20 years of practice: users: 261/417, 62.6% vs non-user: 93/107, 86.9%, *P*<.001); fewer worked under a Health Maintenance Organization (users: 347/417, 83.2% vs non-users: 97/107, 90.7%, *P*<.001) and more worked with practice partners (users: 126/417, 30.2% vs non-users: 4/107, 3.7%, *P*<.001). Efficiency (379/417, 90.9%) and reduction of medical errors (229/417, 54.9%) were the major enabling factors, while patient-unfriendliness (58/107, 54.2%) and limited consultation time (54/107, 50.5%) were the most commonly reported hindering factors. The key functions of computer software among eMR users consisted of electronic patient registration system (376/417, 90.2%), drug dispensing system (328/417, 78.7%) and electronic drug labels (296/417, 71.0%). SoftLink Clinic Solution was the most popular vendor (160/417, 38.4%).

**Conclusions:**

These findings identified several physician groups who should be targeted for more assistance on eMR installation and its adoption. Future studies should address the barriers of using Internet-based eMR to enhance its adoption.

## Introduction

### Background

The introduction of Internet-based information technology (IT) into the health care system is widely perceived as a significant step to improve the quality of services provided by health care institutions [1-5]. As a result, transition of paper medical records to electronic ones is becoming more common in health care systems around the globe [6-9]. These electronic patient records are established in a real-time system with various functions, including instantaneous sharing of patients’ medical history by different health care providers [10]. The delivery of high quality medical services to patients could be much enhanced by reducing medical errors and facilitating more efficient communication among health care professionals by eMR use [9,11]. It becomes a global trend for such electronic systems to be implemented in health care institutions, of either a regional or national scale worldwide [12,13]. Apart from the United States and the United Kingdom, Asia-Pacific countries like Japan, Taiwan, and Singapore also followed this trend and have initiated the development of the computerized system [13]. Although the benefits brought by eMR are substantial, the adoption levels of eMR among these countries are relatively low. The major barriers include the high cost of the hardware and software systems, concerns over the required technological expertise, inertia among physicians, and also lack of government support to bring about changes [9,12].

Hong Kong is one of the most densely populated cities in the world. In order to meet the rising demand for high quality health care, the clinical management system (CMS) developed by the Hospital Authority (HA) was implemented in the public hospitals to allow clinicians timely access to electronic clinical information. It relied exclusively on the Internet as a significant conduit to medical data access. Since 1999, the electronic patient record (HA ePR) was developed to bring information from different modules of CMS into one standardized repository, offering a clinician-friendly interface to access a longitudinal, lifelong patient record [14]. In 2010, there have already been 8 million patient records, 1 million annual admissions, 13 million ambulatory visits, 2 tegabytes ePR data volume, 4 tegabytes ePR images, and 750 million ePR laboratory records in the HA database. The ePR represents one of the most important systems in HA as it consists of more than 12,000 users, 90,000 patients, 2 million transactions via the CMS, as well as 300,000 ePR transactions on a daily basis. The presence of Internet access and high clinician acceptance and utilization are crucial for the Internet-based HA ePR to act as an essential clinical and management tool in clinical practice. These are necessary conditions for transition of paper records to electronic ones [15]. In order to further enhance the benefits brought by HA ePR, the Hong Kong Special Administrative Region government has started the development of a territory-wide Internet-based computerized system—the electronic Health Record (eHR) Sharing System in Hong Kong, which allowed the physicians from both private and public sectors to share patient information. Since currently the private sector provides a significant portion of primary care in Hong Kong, the engagement of clinicians in the private sector becomes one of the key success factors for proper functioning of the system. There is an urgent need for evaluation of the adoption level of the eMR system among private physicians as this provides important information for health informaticians and policy makers to plan future strategies to revamp and enhance the Internet-based eHR sharing.

### Objectives

The objectives of this study were (1) to measure the level of, and factors associated with, the adoption of the eMR system among physicians working in the private sector of Hong Kong, (2) to explore the enabling and hindering factors of the use of eMR, and (3) to evaluate the key functions of eMR and the popularity of electronic systems and vendors used by these private practitioners.

## Methods

### Survey Instruments

A questionnaire was designed and drafted by an academic family physician (MCS) with reference to literature tailor-made to the local context of primary health care in Hong Kong. These questions were face-validated by a panel of epidemiologists, family physicians, informaticians, and academic professors in public health. The questionnaires were then pilot-tested among 15 private practitioners randomly selected from the registry of private practitioners who were honorary tutors of the School of Public Health and Primary Care, Chinese University of Hong Kong (CUHK), and subsequent amendments made according to their recommendations. This study was approved by the Survey and Behavioral Research Ethics Committee of CUHK.

### Target Population and Sampling Methodology

The target population consists of all registered practitioners in Hong Kong working in the private sector. We identified the following sources to trace the contact information of these private practitioners: (1) the Hong Kong Doctors’ website of the Hong Kong Medical Association (HKMA) for the public (n=2464), (2) a list of clinical tutors working in the private sector, carrying an honorary teaching appointment in the School of Public Health and Primary Care of the Chinese University of Hong Kong (n=149), (3) a research database containing the contact details of previous collaborating private practitioners who consented to disclose their contact information for future research (n=247), (4) private doctors’ list from a medical insurance company (Bupa) and members of the Association of Private Medical Specialist (APMS) (n=760), (5) Hong Kong Doctors’ networks in different districts (n=86), and (6) site visits to clinics of various buildings with high concentration of doctors (n=618). We established a central practitioner registry consisting of all registered doctors currently practicing in the private sector from the above sources (N=4405).

We assumed a desired precision level of 5% and the proportion of private practitioners having computerized systems in their clinics being 86% according to a survey conducted by the HKMA in 2006 [11]. Using N=4p(1-p)/(precision)^2^ (where p=proportion of private practitioners who used computers), the minimum sample size was estimated at 193. However, since the use of eMR by some physicians in their clinics might change with time, we used a hypothetical proportion of 50% which would yield the largest sample size, leading to an estimated N=400. Owing to the relatively low response rate of medical doctors to surveys sent (398/6772, 5.88%) in the aforementioned study by the HKMA [11], we decided to send invitations to all private practitioners in our central registry to secure larger sample size. In addition, to increase response rate, we conducted (1) clinic visits to various buildings with high concentration of doctors, (2) visits to sessions awarding Continuous Medical Education (CME) points to the attending physicians hosted by some doctors’ networks with permissions from the seminar organizers, (3) invitations to the chairmen of the private doctors’ Networks in the New Territories West Private Practitioner Network and the Taipo Doctors’ Network, and (4) Invitations to doctors who are chairmen of larger-scale Health Maintenance Organizations (HMOs) to disseminate the surveys to their practice doctors.

### Data Collection

Invitations were sent via faxlines, emails, post with return postage included, site visits, and visits to CME seminars. All surveys were self-administered. Survey invitations were conducted through all these contact channels for each registered doctor identified in our central registry. Hence there may exist multiple invitations to one single practitioner and we checked each returned survey for potential duplication. Up to three telephone or email reminders were sent to the participant physicians to encourage more responses. In addition, we conducted 618 clinic visits to buildings with high concentration of medical doctors and visited two CME seminars (on April 15^th^-16^th^, 2010).

For each survey returned, we checked for the presence of consent signature, full name of the doctor as appeared in the first page of the invitation letter, as well as the completeness of the questionnaires. To ensure confidentiality and anonymity, the first page with doctors’ identity was detached from the survey and each questionnaire was assigned a survey number as a unique identifier by one researcher. Another researcher who collected and entered the data was therefore blinded to the identities of the participant physicians.

### Statistical Analysis

The Statistical Package for Social Sciences version 16.0 (Chicago, Illinois) was used for all data entry and analyses. The major outcome variable was the proportion of private practitioners who used computers in their clinics. We performed descriptive analyses for all survey items. The eMR users and non-users were compared according to their demographic characteristics and practice information using chi-square tests of independence and student’s *t* tests for categorical and continuous variables, respectively. To account for the potential sampling bias due to different invitation methodology (clinic site visits and invitations via practice or network chairmen vs usual faxline /email/ postal invitations), we compared the two groups of participants with regard to their demographic and practice characteristics to detect any heterogeneity. All *P* values less than or equal to .05 were regarded as statistically significant.

## Results

### Participant Characteristics

We received a total of 524 completed surveys via fax, email, postal returns, and on-site collections in clinics and CME seminar venues, giving a response rate of 11.90% (524/4405). The mean age of the study participants was 51.11 years (SD 11.8). Approximately 80.3% (421/524) were male physicians (Table 1). The majority had practice experience of more than 20 years (354/524, 68.9%), and was working under a HMO (444/524, 84.7%). Most were engaged in solo practice (379/524, 72.3%), and possessed specialist qualifications (318/524, 60.7%) recognized by the Hong Kong Academy of Medicine. The survey participants were mainly general practitioners or family physicians (218/524, 41.6%), followed by medical internists (68/524, 13.0%), and surgeons (67/524, 12.8%).

### Profiles of eMR Users vs Non-Users

We analyzed the difference in the characteristics between eMR users (ie, those private practitioners who adopted any electronic computer system for medical consultations in their clinics) and the non-users. Among these private doctors, 417 (79.6%) used computerized systems in their clinics for consultations (Table 1). The adoption levels among family medicine specialists and general practitioners were 83.3% (61/73) and 76.5% (111/145), respectively. The proportions of specialists (who acquired a specialist fellowship recognized by the Hong Kong Academy of Medicine) and non-specialists using eMR were 81.0% and 79.1%, respectively (*P*=.690). They used computers in their clinics for an average of 7.2 years (SD 5.7 years). The eMR users were significantly younger (users: 48.4 years SD 10.6 years vs non-users: 61.7 years SD 10.2 years, *P*<.001) and consisted of a higher proportion of female physicians (users: 80/417, 19.2% vs non-users: 14/107, 13.1%, *P*=.013) as compared with the non-users. The users had less clinical experience (with more than 20 years of practice: users: 261/417, 62.6% vs non-user: 93/107, 86.9%, *P*<.001), and a lower proportion worked under a HMO (users: 347/417, 83.2% vs non-users: 97/107, 90.7%, *P*<.001). There were no statistically significant differences between the users and non-users with regard to their training status (*P*=.105) and clinical specialty (*P*=.617).

**Table 1:** Participant characteristics (N=524).^a^

		Overall (N=524)	eMR^b^ users (n=417)	Non-users (n=107)	*P* value
		n (%)	n (%)	n (%)	n (%)
Age in years, mean (SD)		51.11 (11.8)	48.44 (10.6)	61.72 (10.2)	<.001
**Gender**					
	Male	421 (80.3)	333 (79.9)	88 (82.2)	.013
	Female	94 (17.9)	80 (19.2)	14 (13.1)	
**Practice experience in years**					
	Male, practice experience 0-20 yrs	125 (24.3)	119 (28.5)	6 (5.6)	<.001
	Male, practice experience >20 yrs	294 (57.2)	213 (51.1)	81 (75.7)	
	Female, practice experience 0-20 yrs	33 (6.4)	32 (7.7)	1 (0.1)	.024
	Female, practice experience >20 yrs	60 (11.7)	48 (11.5)	12 (11.2)	
Practice Setting: Health Maintenance Organization		444 (84.7)	347 (83.2)	97 (90.7)	<.001
**Type of practice**					
	Solo	379 (72.3)	283 (67.9)	96 (89.7)	<.001
	With partners	130 (24.8)	126 (30.2)	4 (3.7)	
**Training status**					
	None	150 (28.6)	111 (26.6)	39 (36.4)	.105
	Current or completed Basic training	29 (5.5)	25 (6.0)	4 (3.7)	
	Current or completed higher training	24 (4.6)	22 (5.3)	2 (1.9)	
	Academy Fellow	318 (60.7)	257 (61.6)	61 (57.0)	
**Specialty**					
	Emergency medicine	3 (0.6)	3 (0.7)	0 (0.0)	.617
	Community Medicine	2 (0.4)	2 (0.5)	0 (0.0)	
	Otorhinolaryngology	9 (1.7)	7 (1.7)	2 (1.9)	
	Family Medicine (specialist)	73 (13.9)	61 (14.6)	12 (11.2)	
	General Practice (non-specialist)	145 (27.7)	111 (26.6)	34 (31.8)	
	Obstetrics and Gynaecology	37 (7.1)	28 (6.7)	9 (8.4)	
	Anaesthesiology	4 (0.8)	2 (0.5)	2 (1.9)	
	Ophthalmology	19 (3.6)	19 (4.6)	0 (0.0)	
	General Medicine	68 (13.0)	53 (12.7)	15 (14.0)	
	Orthopedics	31 (5.9)	23 (5.5)	8 (7.5)	
	Pediatrics	39 (7.4)	34 (8.2)	5 (4.7)	
	Psychiatry	9 (1.7)	7 (1.7)	2 (1.9)	
	Radiology	8 (1.5)	8 (1.9)	0 (0.0)	
	Surgery	67 (12.8)	55 (13.2)	12 (11.2)	

^a^Some figures did not add up to 100% due to missing values for some variables.

^b^eMR: electronic medical record

### Reasons for Using or Not Using Computerized Systems Among Private Practitioners

**Figure 1:**
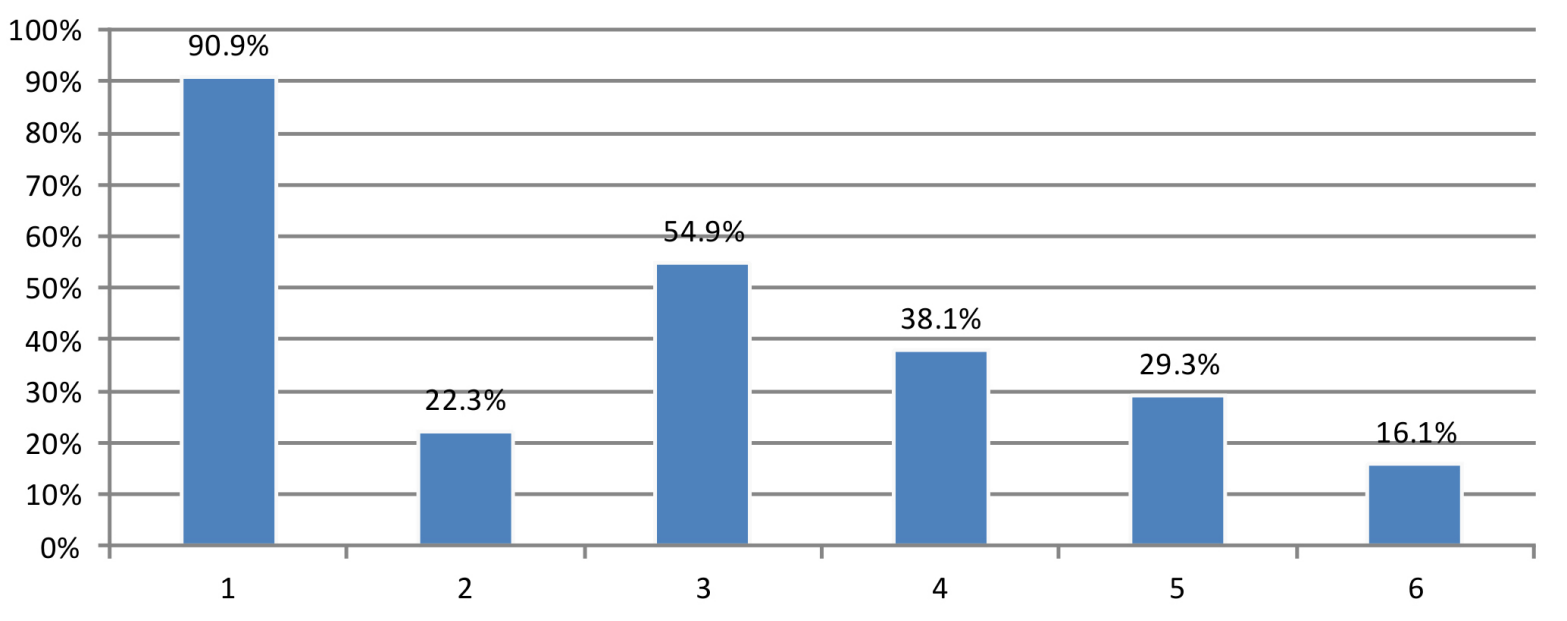
Reasons for using computerized systems in clinics. x-axis: 1=Offer more efficient service; 2=Ability to share patient information in public sector; 3=Reduce medical errors; 4=Eliminate need to store paper records; 5=Eliminate illegibility of my practice partners; 6=Others.

Among the 417 eMR users, the majority perceived efficiency of computerized systems (379/417, 90.9%) as the reason of using computers in their clinics (Figure 1). The other major reasons for using computerized systems included “their ability to reduce medical errors” (229/417, 54.9%), “eliminate the need to store paper records” (159/417, 38.1%), and followed by “eliminate illegibility of practice partners” (122/417, 29.3%). A relatively low proportion of participant physicians used computers due to their “ability to share patient information in the public sector” (93/417, 22.3%).

**Figure 2:**
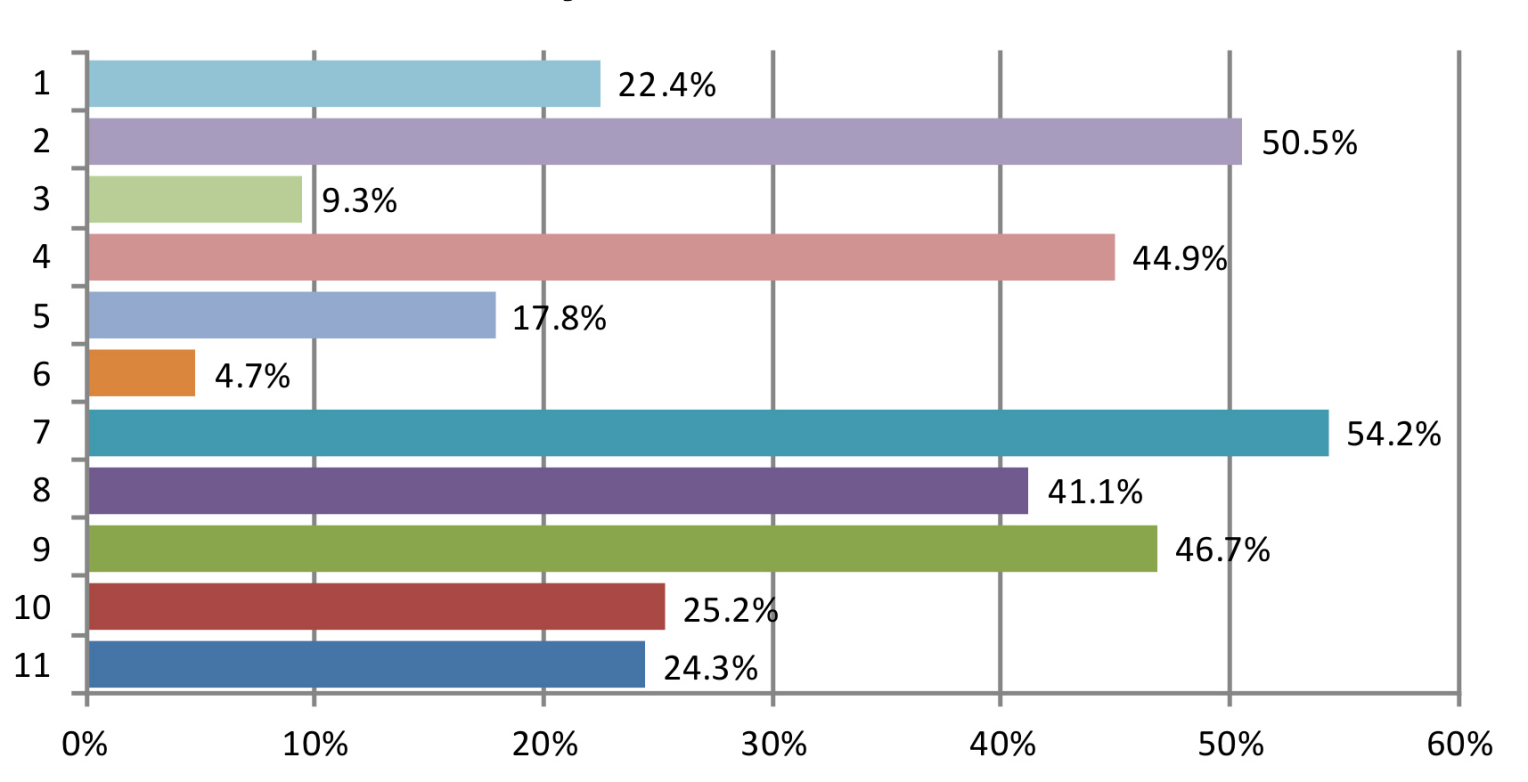
Reasons for not using computerized systems in clinics. x-axis: 1=Cost concerns (Setup/ maintenance); 2=Computer use is more time-consuming; 3=Not supported by the practice partners/ practice organization; 4=Concerns on data migration from paper to system; 5=Insufficient space for computer installation; 6=System not support Chinese language; 7=Not patient-friendly during consultations. 8. Inconvenience caused during down-time 9. Lack of technical support 10. Concerns on computer hackers 11. Others.

Turning to the reasons of not using computers among the other 107 physicians, the most frequently chosen responses included “not patient-friendly during consultations” (58/107, 54.2%) and “computer use is more time-consuming” (54/107, 50.5%) (Figure 2). Significant proportions of respondents also perceived the lack of technical support (50/107, 46.7%), concerned about data migration from paper to system (48/107, 44.9%), and worried about inconvenience caused during computer down-time (44/107, 41.1%).

### Key Functions Included by the Computerized System

**Figure 3:**
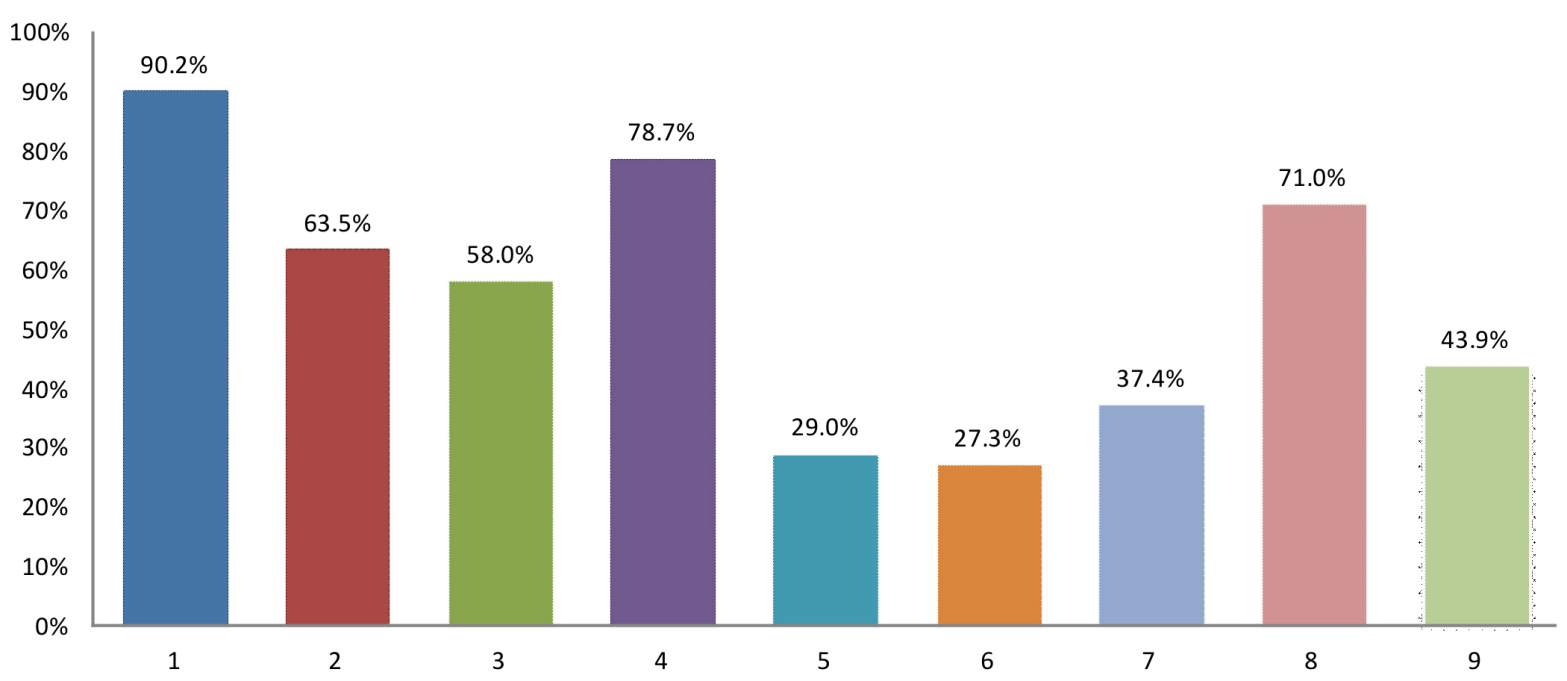
Key functions included by the computerized system. x-axis: 1=Electronic patient registration system; 2=Appointment booking system (e.g. arrangement of next patient visit); 3=Electronic clinical notes (eg, recording of patient history); 4=Dispensing system (eg, printing of prescriptions); 5=Order Entry functions (eg, laboratory, radiological exam order); 6=Picture Archiving and Communication System (PACS); 7=Electronic Health Care Voucher System (eHS); 8=Electronic Drug labels; 9=Public Private Interface-electronic Patient Record (PPI-ePR).

Among the eMR users, electronic patient registration system (376/417, 90.2%) was the most common key functions of the computerized systems (Figure 3). The majority also adopted their computers for drug dispensing which includes the use of dispensing system (328/417, 78.7%) and electronic drug labels (296/417, 71.0%). This is followed by appointment booking system (265/417, 63.5%) and electronic clinical notes (242/417, 58.0%).

### Types of Computer Systems in Current Use

**Figure 4:**
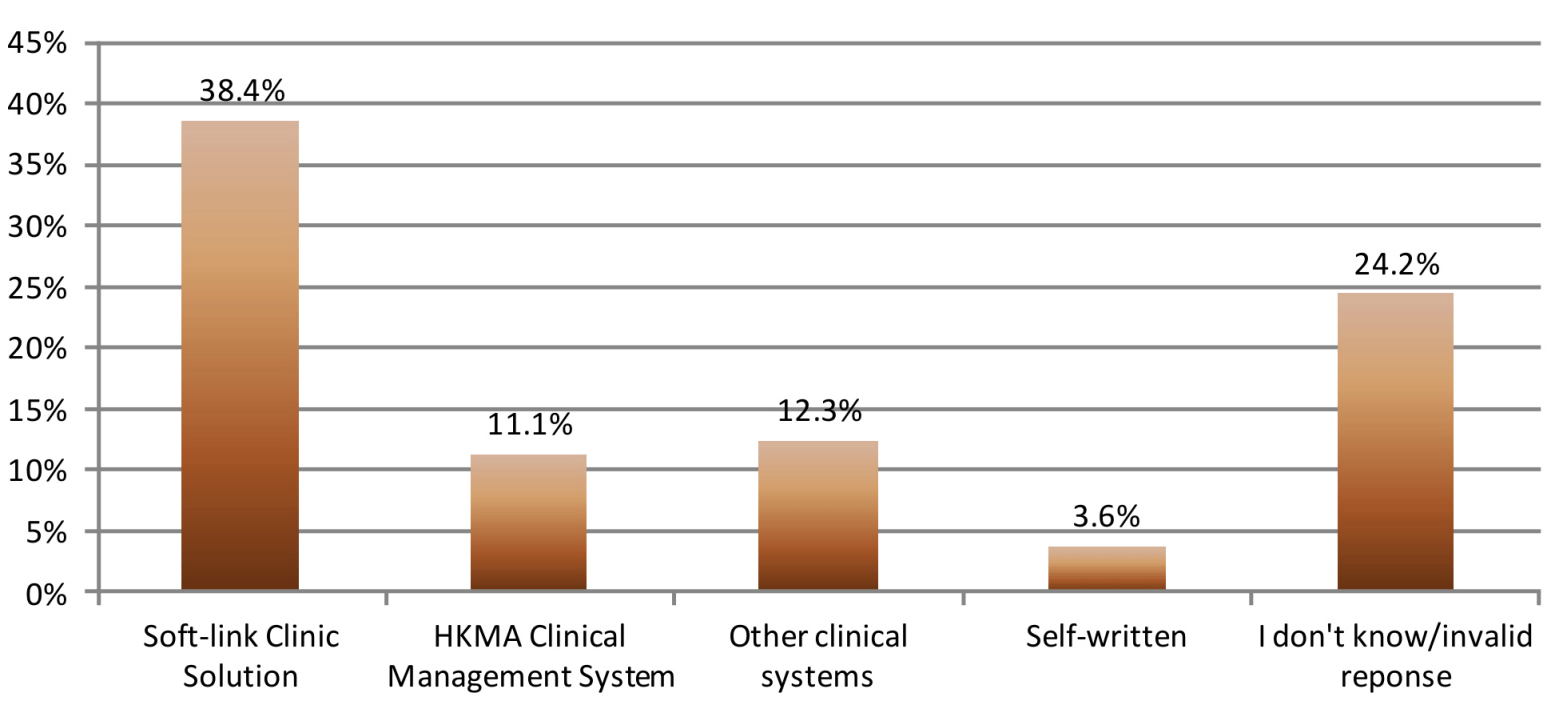
Names of computer system currently in use. Invalid response was defined as naming of computerized systems as Operation Systems (eg, Microsoft Vista) or computer hardware. HKMA: Hong Kong Medical Association.

SoftLink Clinic Solution (160/417, 38.4%) followed by HKMA Clinical Management System 3.0 (CMS 3.0) (46/417, 11.1%) were the most popular computer systems (Figure 4). Around 24.2% (101/417) of private physicians did not know the names of computer systems in use, or gave an invalid response. There was a wide variety of different computer systems adopted by the private physicians.

### Vendors

**Figure 5:**
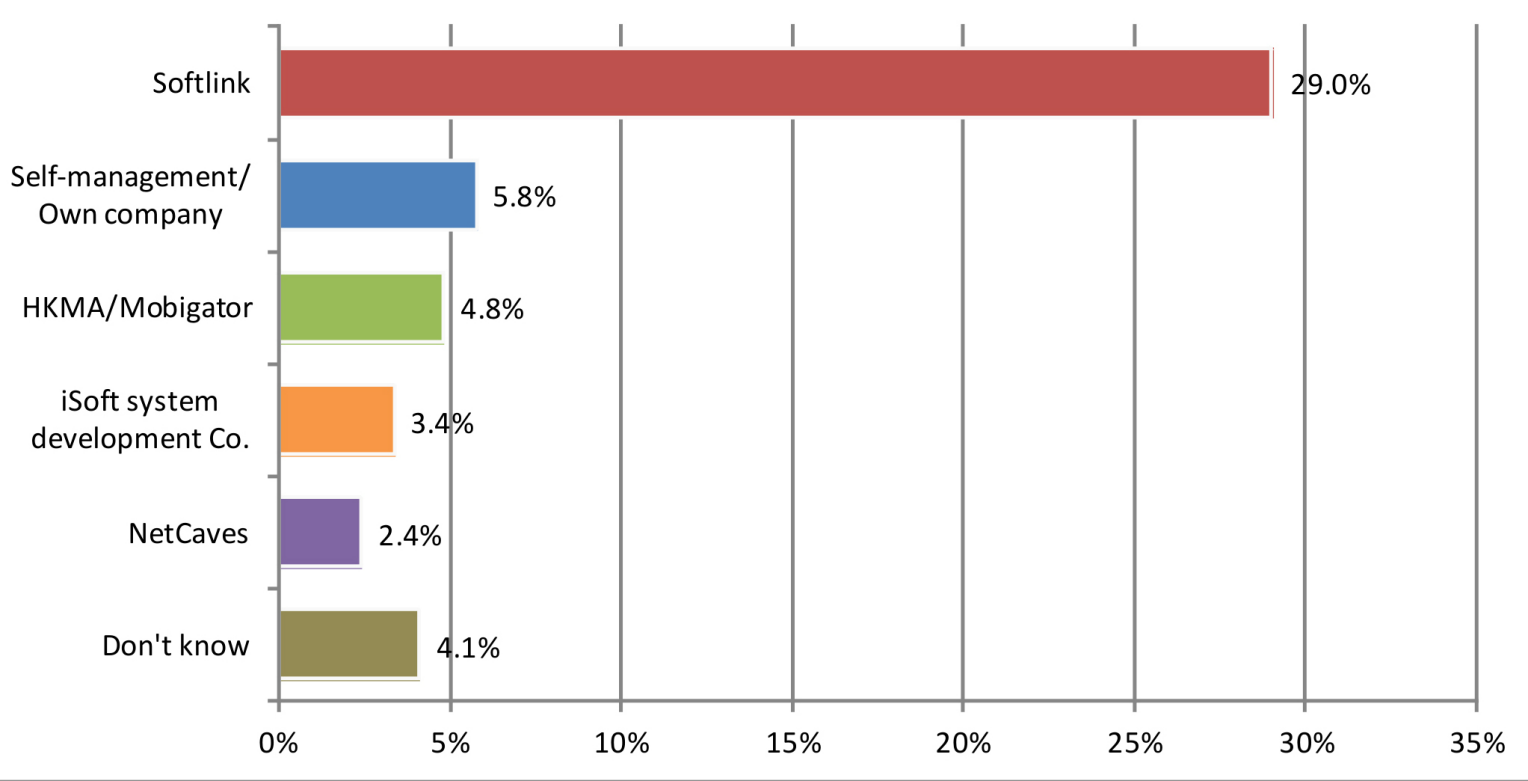
The proportion of participants adopting various vendors. HKMA: Hong Kong Medical Association.

SoftLink (121/417, 29.0%) represented the most frequently chosen vendors among the physicians (Figure 5). The other not uncommonly used vendors included HKMA/Mobigator (20/417, 4.8%), iSoft system development Co (14/417, 3.4%), and NetCaves (10/417, 2.4%). Around 5.8% (24/417) of physicians managed the computers by themselves.

The mean duration of vendor use was 53.9 months (SD 44.0 months) (Table 2). The top reasons for choosing these vendors were introduction by friends (172/417, 41.2%), cost concerns on setup and maintenance (125/417, 30.0%), and reputation of the vendors (125/417, 30.0%). A significant proportion adopted the vendors from the practice management (31/417, 7.4%), while a number of physicians treasured the free service and the continuing system support offered by the vendors (22/417, 5.3%).

### Tests for Sampling Biases

Participants were divided into two groups based on the approach method, where group 1 used clinic site visits and invitations via practice or network chairmen and group 2 used faxline /email/ postal invitations. These groups were tested for heterogeneity with regard to the participants’ demographic and practice characteristics. When group 1 was compared with group 2, there were no differences in age (group 1: mean 55.21 years, SD 15.73 years vs group 2: mean 52.75 years, SD 14.74 years, *P*=.157) and gender (male proportion: group 1: 80.0% vs group 2: 80.6%, *P*=.942) respectively. In addition, we detected no statistically significant differences when years of clinical practice (*P*=.337) and the practice setting (HMO vs non-HMO) (*P*=.105) were tested between the two groups.

**Table 2:** Study participants’ reasons for choosing the current vendors for eMR (N=524).^a^

Reasons for choosing the current vendor	n	%
Cost concerns (setup/maintenance)	125	30.0
Reputation	125	30.0
Introduction by friends	172	41.2
Chosen by practice management	31	7.4
At random	18	4.3
Others	85	20.4

^a^Duration of vendor services: mean 53.85, SD 44.00

## Discussion

### Principal Findings

This study found that among 524 private physicians, 79.6% (417/524) adopted computerized systems in their clinics. The computer users were significantly younger, more were female, possessed less clinical experience, and less worked under an HMO. The major reasons of using computers in their clinics included perceived computer efficiency, reduction of medical errors, elimination of need to store paper records as well as issues related to case note illegibility. The high and similar prevalence of using eMR in the clinical practice of both specialists and non-specialists indicated that a communication culture on sharing patient records through extensive computer network has been established between these 2 groups of physicians as this brings convenience of extracting updated information of patients through eMR during consultation. This was also reflected from their heavy use of electronic patient registration system, dispensing system, and electronic drug labels printing system, which are part of the eMR system. Among the users, the key functions of computerized systems included electronic patient registration and drug dispensing. Among the non-users, the use of computers was regarded by most as patient-unfriendly and time-consuming during clinical consultations and it was quite surprising that the impression of eMR system between eMR and non-eMR users was quite different. Therefore, there may probably be a misunderstanding on the eMR system and further efforts should be made, especially tackling the opinions from the non-users, in order to increase the overall prevalence of using eMR system. SoftLink Clinic Solution was the most frequently used computer system and also vendor. It is a comprehensive software system allowing physicians an easy documentation of electronic medical notes and access to clinical images and laboratory reports of patients. Drug label printout system is also integrated into the system. The preference of the computer system was found to be diversified and this might lead to more adoption of SoftLink Clinic solution over other current choices in the market. The choice of vendors was mostly influenced by friends, setup and maintenance costs, and their reputation.

There is a scarcity of local studies on the adoption levels of eMR in the private sector. To our knowledge, there was only one study conducted by Ho et al [16] who sent 6772 questionnaires to both HKMA members and non-members via the HKMA Circulars in 2006. The response rate was 5.88% (398/6772) and they found that 86% (342/398) used computers in workplace. When enquired about the use of clinic management package in the study by Ho et al, only 43% (171/398) gave a positive response. The higher proportion of physicians using computerized systems in the clinics as reported in this study (317/398, 79.6%) was however not directly comparable to their studies as we used a different methodology and a broader definition of computers was referred to (ie, we included any electronic health records in addition to computer management system).

The level of computer adoption in this study is high (417/524, 79.6%). When a sub-analysis of Ho’s study [16] was conducted where only private physicians were included, the adoption level was 81.7% (192/235), a figure similar to the present study. However, as the private sector provides more than 70% of primary care in Hong Kong, and that we do not have data on the compatibility of the current use of computerized systems to share health records with the public sector, there seems to have a further room to enhance computer use among private practitioners. In this connection, the major reasons of computer use, namely their efficiency, ability of reducing medical errors and case note illegibility, as well as their capability to eliminate the needs for medical record storage, should be promulgated to the eMR non-users. On the other hand, the issues of patient-unfriendliness and the perceived time-consuming nature associated with computer use should be addressed [17,18]. Besides, there have been studies reporting that non-users might perceive threat to their professional autonomy by eMR, including loss of control over their clinical work and restrictions of their clinical freedom [19]. The eMR initiatives need to demonstrate the unique advantages of adopting computerized systems in the clinics by promoting the different attractive functions possessed by the current computer softwares. More technical assistance is warranted for installation, maintenance and support of computers for private practitioners as this was quoted by many as a hindering factor of computer use [20-23].

The low proportion of eMR users whose reason to use computers in their clinics was to share patient information with the public sector might reflect their low intention to do so. This is echoed by the relatively low proportion of computer users having Public Private Interface-electronic Patient Record (PPI-ePR) Sharing Pilot Project, which is a pilot programme allowing sharing patients’ electronic records among the public and private sectors, as the key functions of their installed systems. Many of the motivators to use computers identified in this study were related to efficiency and convenience of clinical practice instead of information sharing between the public and private sector. The importance of sharing patients’ records between the two sectors should be more emphasized among private practitioners. Extra personal incentives could also be provided to encourage the use of the eMR system [24].

The friends of the private physicians, many might well be medical colleagues, were found to be more influential on the choice of vendors than the set-up and maintenance cost required for the eMR system and the reputation of vendors. This reflected that the costs of the eMR system might not be a heavy burden for the physicians and recommendations from other physicians will be a good initiation for the use of eMR system in the clinical practice. Seminars could be organized where colleagues of the same specialty share their positive experience of using eMR in their clinics tailor-made to their clientele for the eMR non-users. In addition, as free services including computer setup and ongoing system support have been raised as an important consideration by a number of physicians who were currently using eMR, initiatives on provision of such services at low costs could be considered for the non-users to incentivize their adoption of computerized systems in their clinics.

### Limitations

This study included more than 500 surveys and the precision achieved is higher than the traditionally used 5%. However, some of its limitations should be mentioned. First, the response rate was modest (524/4,405, 11.9%) although previous studies among physicians yielded even lower response rates at the levels of approximately 5% (398/6772). There existed non-response bias, and it is conceivable that those without computers might be less interested to participate in the survey. Second, we do not have the contact information of all private practitioners in Hong Kong. In addition, the sampling frame is a mix between the usual invitation group: by postal mailing, faxline, email, and the on-site visit group: clinic visits and survey invitations during CME seminar, thus introducing sampling bias. However, this sampling bias should be regarded as minimal as shown by our separate analysis where no differences in the demographic and practice characteristics between the two groups were detected. Last, the surveys received names of computers and vendors interpreted by the participant physicians differently. All programs or applications must run on an Operation System as a platform. For instance, Microsoft Vista is an Operation system. Clinic management system is a generic name for the software used for clinic management (including clinic solution, WinMed, HKMA CME 2.0 and HKMA CMS 3.0 etc) and the Clinic Solution is one of the Clinic management systems. The Clinic Solution is the CMS developed by the SoftLink, hence SoftLink is the name of the company but not a software. It is not expected that the participant physicians could provide details of computerized systems and vendors at these different levels in details, and hence a distinction could not be made here due to the lack of additional information.

### Conclusions

In summary, this survey provided a cross-sectional description of the current adoption of eMR and their vendors in the private sector, and depicted the major reasons of their use and non-use. Based on the demographic characteristics of the non-users (more likely older, male physicians, more practice experience, work under HMO, and solo practice), knowledge of eMR installation and maintenance should be conveyed to these physician groups. The competitive advantages of eMR use in clinics, namely their efficiency and convenience favorable to the practice, should be shared with the non-users by the current users, preferably having similar clientele. The major reasons of not using eMR, among the non-users should be further addressed and tackled with. These include strategies to make computer use in clinics equally patient-friendly as compared to not using computers, as well as addressing the possible misperception that computer adoption is time-consuming. More technical supports, including lower cost computer setup and system support services, should be made readily available for the current non-users to remove barriers of eMR use. Future studies should be conducted to capture more data from practices not reachable due to absence of contact information. Site visits may lead to a high response rate and future research should consider further survey services by clinic visits.

## Abbreviations

APMS: the Association of Private Medical Specialist
CME: Continuous Medical Education
CMS: clinical management system
CUHK: Chinese University of Hong Kong
eHR: electronic Health Record
eMR: electronic medical record
ePR: electronic patient record
HA: Hospital Authority
HKMA: Hong Kong Medical Association
HMO: Health Maintenance Organizations
IT: information technology
